# Concordance of HER2-low scoring in breast carcinoma among expert pathologists in the United Kingdom and the republic of Ireland –on behalf of the UK national coordinating committee for breast pathology

**DOI:** 10.1016/j.breast.2023.06.005

**Published:** 2023-06-27

**Authors:** Mohamed Zaakouk, Cecily Quinn, Elena Provenzano, Clinton Boyd, Grace Callagy, Soha Elsheikh, Joe Flint, Rebecca Millican-Slater, Anu Gunavardhan, Yasmeen Mir, Purnima Makhija, Silvana Di Palma, Susan Pritchard, Bruce Tanchel, Emad Rakha, Nehal M. Atallah, Andrew H.S. Lee, Sarah Pinder, Abeer M. Shaaban

**Affiliations:** a Institute of Cancer and Genomic Sciences, University of Birmingham, Birmingham, UK; b Cancer Pathology, National Cancer Institute, Cairo University, Cairo, Egypt; c Department of Histopathology, St Vincent's University Hospital, Elm Park, Ireland; d UCD School of Medicine, Dublin, Ireland; e Addenbrookes Hospital and NIHR Cambridge Biomedical Research Centre, Cambridge, UK; f Department of Histopathology, Addenbrookes Hospital, Cambridge, UK; g Histopathology, Belfast Health and Social Care Trust, Belfast, UK; h Discipline of Pathology, University of Galway, School of Medicine, Lambe Institute for Translational Research, Galway, Ireland; i Department of Cellular Pathology, Royal Free Hospital, London, UK; j Research Department of Pathology, University College London, Cancer Institute, London, UK; k Birmingham Tissue Analytics, University of Birmingham, UK; l Department of Cellular Pathology, St James's University Hospital, Leeds, UK; m Department of Histopathology, Glan Clwyd Hospital Betsi Cadwaladr University Health Board, Bodelwyddan, UK; n Pathology, Liverpool University Hospitals Foundation Trust, Liverpool, UK; o Pathology, Barts Health NHS Trust, London, UK; p Cellular Pathology Department, Royal Surrey Hospital NHS Foundation Trust, Guildford, UK; q Pathology, Wythenshawe Hospital Manchester Foundation Trust, Manchester, UK; r Cellular Pathology, Heart of England NHS Foundation Trust, Birmingham, UK; s Histopathology Department, Nottingham University Hospitals NHS Trust, City Hospital Campus, Nottingham, UK; t Department of Pathology, Faculty of Medicine, Menoufia University, Egypt; u School of Cancer & Pharmaceutical Sciences, Kings College London, London, UK; v Cellular Pathology, Queen Elizabeth Hospital Birmingham, Birmingham, UK

**Keywords:** Breast cancer, HER2-Low, HER2-Ultralow, Consistency, Concordance, Immunohistochemistry

## Abstract

**Background:**

Recent clinical evidence showed that breast cancer with low HER2 expression levels responded to trastuzumab deruxtecan therapy. The HER2-low cancers comprise immunohistochemistry (IHC) score 1+ and 2+ ISH non-amplified tumours, currently classified as HER2 negative. Little data exists on the reproducibility of pathologists reporting of HER2-low cancer.

**Patient and methods:**

Sixteen expert pathologists of the UK National Coordinating Committee for Breast Pathology scored 50 digitally scanned HER2 IHC slides. The overall level of agreement, Fleiss multiple-rater kappa statistics and Cohen's Kappa were calculated. Cases with low concordance were re-scored by the same pathologists after a washout period.

**Results:**

Absolute agreement was achieved in 6% of cases, all of which scored 3+. Poor agreement was found in 5/50 (10%) of cases. This was due to heterogeneous HER2 expression, cytoplasmic staining and low expression spanning the 10% cut-off value. Highest concordance (86%) was achieved when scores were clustered as 0 versus others. Improvement in kappa of overall agreement was achieved when scores 1+ and 2+ were combined. Inter-observer agreement was moderate to substantial in the whole cohort but fair to moderate in the HER2-low group. Similarly, consensus-observer agreement was substantial to almost perfect in the whole cohort and moderate to substantial in the HER2-low group.

**Conclusion:**

HER2-low breast cancer suffers from lower concordance among expert pathologists. While most cases can reproducibly be classified, a small proportion (10%) remained challenging. Refining the criteria for reporting and consensus scoring will help select appropriate patients for targeted therapy.

## Highlights

• Concordance of HER2 low scoring using digital whole slide images was assessed among 16 expert pathologists.
• 10% of cases were challenging to classify; all were of the HER2 low and ultralow.
• Heterogeneity, cytoplasmic expression and percentage expression around 10% were reasons for discordance.
• Clustering of categories (0 versus others) resulted in the highest concordance levels.

## 1 Introduction

Since the introduction of adjuvant trastuzumab anti-HER2 therapy and the subsequent development of several further anti- HER2 agents, accurate identification of patients with HER2 positive breast carcinoma has been essential to ensure appropriate personalised management of their disease [1]. This requires diagnostic tests that can correctly identify HER2 positive breast cancers. For the past 2 decades, a binary algorithm has been used that classifies breast cancer into HER2 positive (immunohistochemistry (IHC) scores 3+ and/or 2+ in situ hybridisation (ISH) amplified) and HER2 negative (IHC scores 0,1 or 2+ ISH non amplified) [2,3].

Recently, it was recognized that a group of HER2-low expressing tumours responded to trastuzumab deruxtecan (T-DXd) therapy; an antibody drug conjugate (ADC) that affects cancer cells expressing small quantities of HER2 protein and those surrounding them via a by-stander effect. DESTINY-Breast 04 was the first open label randomised controlled phase 3 trial for T-DXd for advanced and all metastatic HER2-low breast cancer including both hormonal receptor positive (n = 494, 88.7%) and negative (n = 63, 11.3%) tumours. Both progression free survival and overall survival were significantly longer in the T-DXd treated group compared with physician choice (9.9 versus 5.1 months and 23.4 versus 16.8 months, p < 0.001 for both) [4]. Based on the trial findings, the Food and Drug Administration (FDA) approved T-DXd for the treatment of patients with unresectable or metastatic HER2-low breast cancer. The drug has subsequently been approved by the European Medicines Agency as the first monotherapy for treating metastatic HER2-low breast cancer [5]. In the UK, T-DXd is currently licenced as monotherapy for the treatment of patients with unresectable or metastatic HER2+ breast cancer who have received one or more prior anti-HER2-based regimens [6] and its use for the same FDA approved indication is being considered by the National Institute for Health and Care Excellence (NICE).

HER2-low tumours (IHC score 1+ and/or 2+, ISH non-amplified) are presently classified as HER2 negative and include both luminal (oestrogen receptor positive) and triple negative breast cancer (TNBC). When the value of HER2-low breast cancer becomes widely applicable, clinical decision-making will require a move away from the binary paradigm for HER2 scoring. However, there are minimal data regarding the reliability of HER2-low scoring among pathologists and this category is not currently separated from the HER2 negative cancers in daily practice. The recently updated UK HER2 reporting guidelines [7] and the updated American Society of Clinical Oncology–College of American Pathologists Guideline Update [8] have recognized the importance of distinguishing the HER2-low cancers to inform therapy with trastuzumab deruxtecan. In a recent study of 105 HER2 non-amplified breast carcinomas scored by 16 pathologists, Baez-Navarro et al., showed moderate overall concordance [9]. Furthermore, Scott et al., in 2021 showed significant discordance in the reported HER2 status between local and central review laboratories with concordance rates of 70.8% and 40% for HER2 1+ and HER2 2+ groups respectively [10].

The present study aimed to assess the inter-observer concordance in the reporting of HER2-low and ultralow breast carcinoma among expert breast pathologists to assess the accuracy of identifying this group of tumours, highlight areas of reporting difficulty and provide suggestions for improving analytical consistency.

## 2 Materials & methods

50 invasive breast cancer core biopsies, from routine clinical practice, enriched for HER2 scores 0, 1+ and 2+ categories were selected from the archives of a single large UK institution (Queen Elizabeth Hospital Birmingham). The department is a reference centre for HER2 immunohistochemistry and FISH testing, performing approximately 4400 HER2 immunohistochemistry tests and 1200 HER2 Fluorescent in situ hybridisation (FISH) tests per annum. All slides were stained using the Ventana 4B5 assay on the Ventana Autostainer ((Ventana BenchMark Ultra, Roche, Indianapolis, USA) following the manufacturer's instructions. Each slide included 3 breast cancer tissue controls of HER2 scores 0, 2+ and 3+ as per the UK National External Quality Assurance Scheme (NEQAS) recommendations [11]. Paired H&E and HER2 IHC slides were reviewed prior to scanning to ensure adequate amount of tumour tissue and optimal quality of staining. Slides were then digitally scanned using a Leica Aperio AT2 slide scanner (Leica Biosystems Imaging, California, USA) at ×40 magnification and uploaded to the University of Birmingham digital platform via a secure link: https://eslidepath.bham.ac.uk. Each participant was provided with secure access to the digital platform using a unique username and password.

**Fig. 1:**
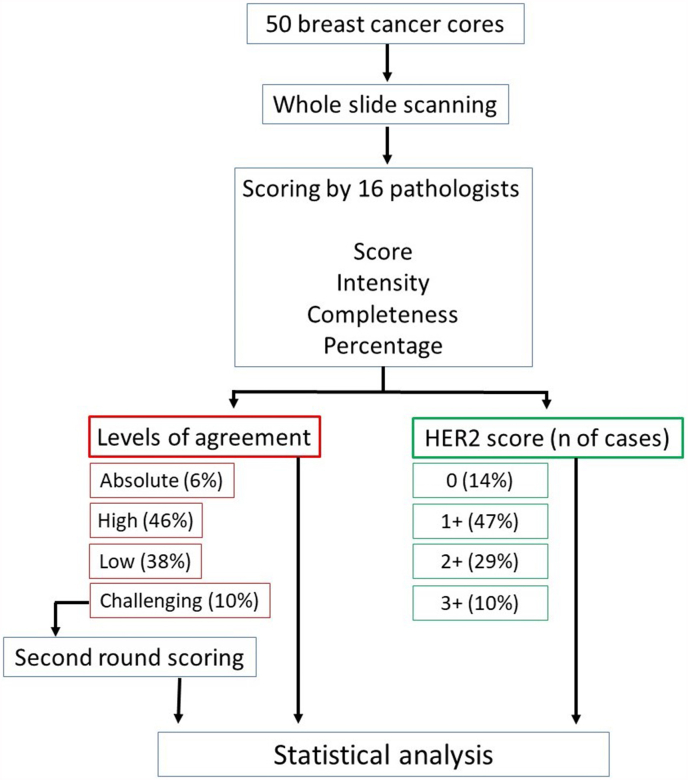
Flow chart of the study design.

The study cases were scored by 16 pathologist members of the UK National Coordinating Committee for Breast Pathology (NCCBP); a steering committee for the UK National Health Service (NHS) Breast Screening Programme and for the Royal College of Pathologists. Membership includes expert breast pathologists acting as professional clinical advisors to the NHS Breast Screening Programme and covering the geography of the UK and Republic of Ireland. In addition to the final HER2 scores of 0, 1+, 2+ or 3+, participants also scored the percentage (<10% or ≥10%), intensity and completeness of membranous expression (complete versus incomplete) to arrive at the final scoring. This method is more detailed and is in line with the algorithm for HER2 scoring in the updated UK HER2 scoring guidelines [7]. Fig. 1 summarizes the study design and Table 1 shows the parameters assessed by each of the scoring pathologists.

**Table 1:** HER2 scoring parameters.

Parameter	Score	Description
HER2 Final score	0	Negative
	1+	Negative (1+)
	2+	Equivocal
	3+	Positive
Intensity	0	None
	1	Faint
	2	Weak
	3	Moderate
	4	Strong
Completeness of membrane staining	0	Incomplete
	1	Complete
Percentage (%)	0	<10
	1	≥10

Data on patients’ demographics, tumour type, grade and hormonal status were collected. The original HER2 scores were obtained from the histopathology reports. The FISH amplification status, for the HER2 IHC score 2+ (equivocal) cases, was also recorded.

## 3 Statistical analysis

Statistical analysis was performed using the SPSS (IBMS) software version 28. For statistical analysis, cases were categorised into whole cohort as well as HER2-low group (HER2 IHC score 1+ or 2+ FISH non-amplified), HER2-ultralow (score 0 with incomplete membrane staining of less than 10%) and HER2 positive (IHC score 3+ or 2+ FISH amplified).

If all participants agreed (100%; 16/16 scorers) this was regarded as absolute agreement (AA). The HER2 preparations with agreement of 12–15 (75–94%) raters were regarded as showing high agreement, those with agreement of 9–11 raters (>50-<75%) were regarded as low agreement level, while those with ≤8 (≤50%) were considered to represent challenging cases (Table 2). In addition to calculating the level of agreement for the 4 standard scoring categories (0, 1+, 2+, 3+), further three clustered scoring categories were used as follows: 0/1 (for IHC scores 0 and 1+), 1/2 (for IHC scores 1+ and 2+) and lastly dividing scores into 0 against all other scores.

**Table 2:**
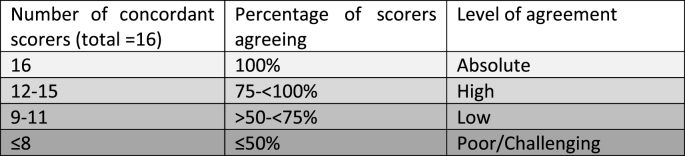
Levels of agreement.

HER2 stained tumours designated as challenging with agreement only among 8 pathologists (50%) were studied and re-scored by all 16 pathologists. Since the level of agreement does not necessarily reflect clinical significance in management of the patients, if the scores were all on the positive or the negative sides, only scores which were significantly different, resulting in potential over or under-treatment, were designated as significant minority scoring.

Fleiss multiple-rater kappa (κ) statistics of inter-observer agreement for scoring cases (as 0, 1+, 2+ and 3+) was calculated. Fleiss' kappa, κ is a measure of inter-rater agreement used to determine the level of agreement between two or more raters when the method of assessment, known as the response variable, is measured on a categorical scale. Fleiss multi-rater kappa was used to measure overall agreement (OA) and agreement on scoring categories, among all the pathologists. Cohen's weighted Kappa was calculated to assess inter-observer agreement between each two of the scoring pathologists as well as between each pathologist and the consensus score. Cohen's weighted kappa was analysed both in the whole cohort and in the HER2-low group. Kappa results are interpreted as follows: values ≤ 0 indicate no agreement and 0.01–0.20: none to slight, 0.21–0.40: fair, 0.41–0.60: moderate, 0.61–0.80: substantial and 0.81–1.00: almost perfect agreement.

For statistical analysis, the consensus score was considered the reference score, upon which cases were classified into HER2 negative, ultralow, low and positive categories.

## 4 Results

### 4.1 Cohort characteristics

The cohort comprised 50 breast tumours. The median age of patients was 58.5 years (IQR 50.75-76.25). The invasive carcinomas were predominantly grade 2 (29/58; 58%). Invasive carcinoma of no special type (NST) was the commonest (41/50; 82%), followed by invasive lobular carcinoma (4/50; 8%).

The original HER2 scoring, designated by the pathologist at the time of reporting, divided tumours into score 0 (20/50; 40%), 1+ (13/50; 26%), score 2+ ‘equivocal’ (12/50; 24%) and 3+ (5/50; 10%). Equivocal cases (score 2+) were subsequently categorised by FISH into non-amplified (10/50; 20%) and amplified (2/50; 4%), Table 3.

**Table 3:** Clinicopathological characteristics of the studied cohort.

Age (yrs)	Median (IQR)	58.50 (50.75: 76.25)		
Grade	1	10/50; 20%		
	2	29/50; 58%		
	3	11/50; 22%		
Histologic Type	NST		41/50; 82%	
	Lobular		4/50; 8%	
	Tubular		2/50; 4%	
	Mucinous		1/50; 2%	
	Metaplastic		1/50; 2%	
	Apocrine		1/50; 2%	
Molecular Type	Luminal		37/50; 74%	
	Her2 positive		8/50; 16%	
	Triple negative		5/50; 10%	
ER	Status		Intensity	
	Negative	7/50; 14%	Absent	7/50; 14%
	Positive	43/50; 86%	Weak	3/50; 6%
			Moderate	0/50; 0%
			Strong	40/50; 80%
PR	Status		Intensity	
	Negative	15/49; 31%	Absent	15/49; 31%
	Positive	34/49; 69%	Weak	1/49; 0.02%
			Moderate	2/49; 0.04%
			Strong	31/49; 68.94%
HER2 Original Score	0	1/50; 2%	No staining	Negative
		19/50; 38%	Staining in <10%	Negative (HER2 ultralow)
	1+	13/50; 26%		Her2-low
	2+	10/50; 20%	FISH Non-Amplified	Her2-Low
		2/50; 4%	FISH Amplified	HER2 Positive
	3+	5/50; 10%		HER2 Positive

### 4.2 Consensus scoring

The 16 pathologists scored 50 cases, with a total of 800 scores recorded. The most common category was 1+ (323/800; 40.40%), followed by 2+ (259/800; 32.38%), 0 (136/800; 17%) and 3+ (82/800; 10.25%). When the consensus score (score agreed by the majority of raters) was considered, the distribution of scores was comparable, where score 1+ was the mostly agreed score (23.5; 47%), followed by 2+ (14.5; 29%), 0 (7; 14%) and 3+ (5; 10%). According to consensus/majority scoring, the 7 tumours scored as 0, were subsequently divided into HER2 negative (1/50; 2%) and HER2 ultralow (6/50; 12%), the latter defined as evident focal staining in less than 10% of cells. Tumours scored as 2+, were divided according to the results of prior FISH testing where available (results not known to the participating pathologists/raters) into FISH amplified (n = 2) and FISH non-amplified (n = 12.5) including a case with no agreement with half the raters scoring it as 2+ and the other half as 1+. The latter group, in addition to cases scored as 1+ (n = 23.5), represented the HER2-low group (n = 36). FISH amplified cases, in addition to cases scored as 3+, represented the HER2 positive tumour group cases (n = 7). The distribution of the 800 scores, submitted by the pathologists and the consensus scores are shown in Table 4.

**Table 4:** Distribution of the scores among raters.

Pathologist	HER2 Scoring Categories					
	0		1+	2+		3+
P1	19		13	13		5
P2	6		18	22		4
P3	3		12	31		4
P4	4		27	13		6
P5	7		14	25		4
P6	5		33	7		5
P7	7		19	19		5
P8	20		17	9		4
P9	15		20	10		5
P10	1		20	20		9
P11	9		22	14		5
P12	19		16	10		5
P13	5		34	7		4
P14	6		24	16		4
P15	2		10	32		6
P16	8		24	11		7
Total (n); %	136 17		323 40.4	259 32.4		82 10.2
Consensus (n); %	7 14		23.5 47	14.5 29		5 10
HER2 Categories	N	UL	LOW		POS	
(n)	1	6	36		7	

N: Her2 negative; UL: HER2 ultralow; LOW: HER2-low; POS: HER2 positive.

### 4.3 Levels of agreement

The percentage of complete agreement (absolute agreement), in which all 16 pathologists assigned tumours to the same IHC score category (0, 1+, 2+ or 3+), was achieved in only 6% (3 of 50), all of which were scored as 3+, Fig. 2A. No absolute agreement was achieved in tumours scored as 0, 1+ or 2+ (Table 5).

**Fig. 2a:**
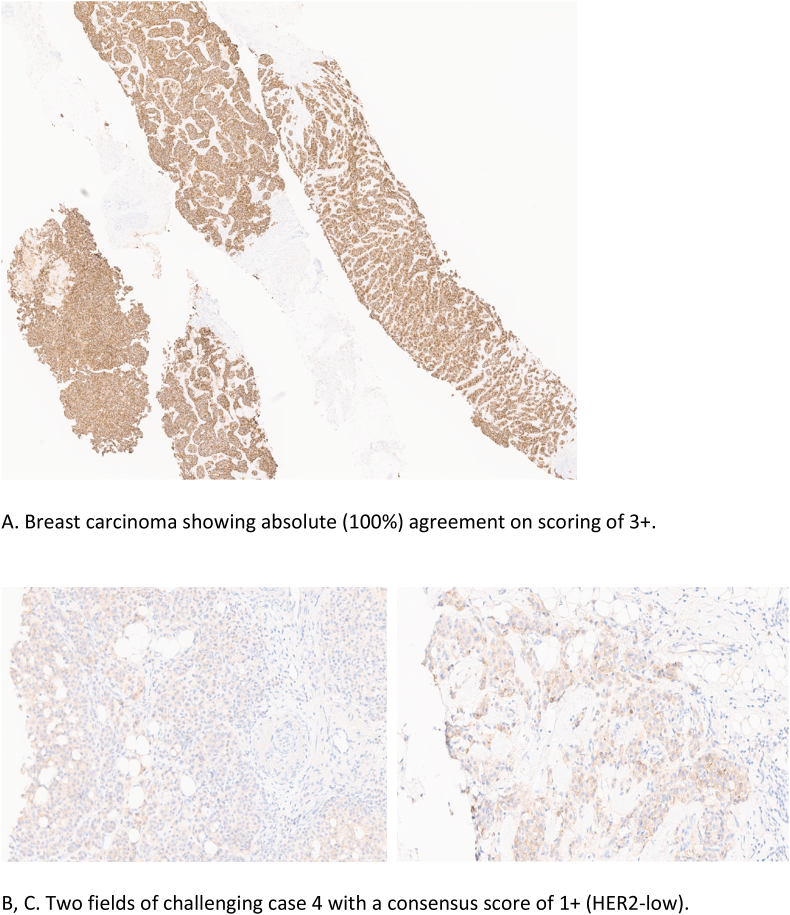
Examples of HER2 immunohistochemical staining and interpretation in the studied cohort. A. Breast carcinoma showing absolute (100%) agreement on scoring of 3+. B, C. Two fields of challenging case 4 with a consensus score of 1+ (HER2-low).

**Table 5:**
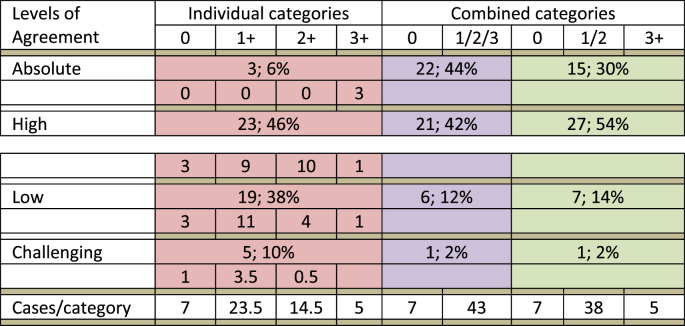
Levels of agreement across standard & clustered categories.

When HER2 scores were combined, the level of agreement increased, the percentage of tumours with absolute and high agreement both increased. The highest level of absolute and high agreement (86%) was achieved when tumours were divided into just 2 categories (score 0 versus others). This was followed by using the combined category (1+/2+) where high and absolute agreement was achieved in 84% of tumours. For the HER2-low category alone, combining scores 1+ and 2+ resulted in an absolute and high agreement in 92% of cases.

## 5 Challenging cases

Overall agreement above 50% (8/16) was seen in the majority of tumours (45/50, 90%) while the remaining tumours (5/50; 10%) had poor agreement. These challenging tumours were all HER2 negative, of luminal molecular type and belonged to the HER2-low (n = 4) and HER2 ultralow (n = 1) groups, Table 6. Using the current HER2 scoring algorithms and clinical management pathways, these tumours would all be classified as HER2 negative and no adverse clinical impact expected. Reasons for discordance included heterogeneous HER2 expression with some focal staining bordering the 10% cut-off leading to difficulties in discerning 0 from the 1+ category. Cytoplasmic staining was also a major contributing factor particularly when prominent and mimicking faint/weak membrane staining, leading to overestimation of HER2 staining Fig. 2a, Fig. 2bB,C, 2D. One case was focally blurred on the digital scanned image resulting in difficulties in scoring.

**Table 6:**
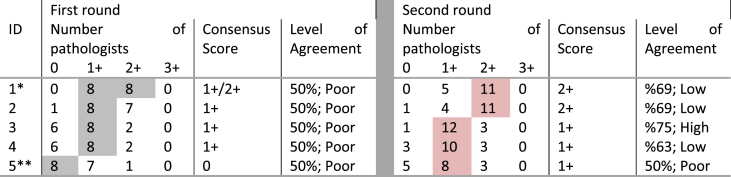
Challenging cases consensus first and second round scoring. *(*1*) Lobular carcinoma; (5*+) Mucinous carcinoma; rest of the cases were of the NST histological type. On the left side of the table; grey-shaded cells represent the number of raters agreeing on scoring (8/16 or lower; 50% denote poor level of agreement). On the right side of the table; pink-shaded cells display number of raters agreeing on a particular score, after re-scoring (8/16 or lower; 50%).

**Fig. 2b:**
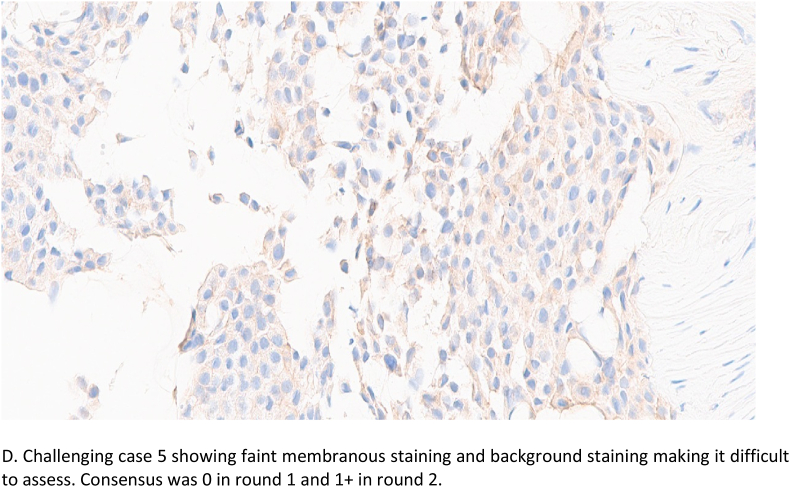
D. Challenging case 5 showing faint membranous staining and background staining making it difficult to assess. Consensus was 0 in round 1 and 1+ in round 2.

The 5 challenging cases were subsequently re-scored (supplementary spreadsheet 1) to assess intra-observer concordance. The distribution of raters’ scores in both rounds is shown in Table 6. The second round scoring resulted in better level of agreement in 4 cases, while agreement remained the same in the fifth case. The level of agreement improved from poor to low and high in 3/5 and 1/5 cases respectively, while remained poor in the fifth case. This latter case was a mucinous carcinoma.

### 5.1 Minority scores of adverse clinical significance

Tumours which showed a difference between each rater's score and the consensus score, potentially resulting in either under or over-treatment and hence inappropriate management, were considered scores of adverse clinical significance. Eight tumours (8/50; 16%) showed such significant minority scoring and were submitted by a total of 6 pathologists (Table 7). These 8 tumours included those designated as negative (n = 3) while the consensus was HER2 positive, and tumours classified as positive (n = 5) with the consensus being negative. One tumour (case 8, Table 7) showed two distinct clones (clustered heterogeneity); a minority 3+ clone and a majority 0 clone with variable interpretation of results (Two pathologists reported as 3+, three reported as 1+ and one as 0, while majority (n = 10) as 2+). Fig. 2c, Fig. 2dE and 2F.

**Table 7:**
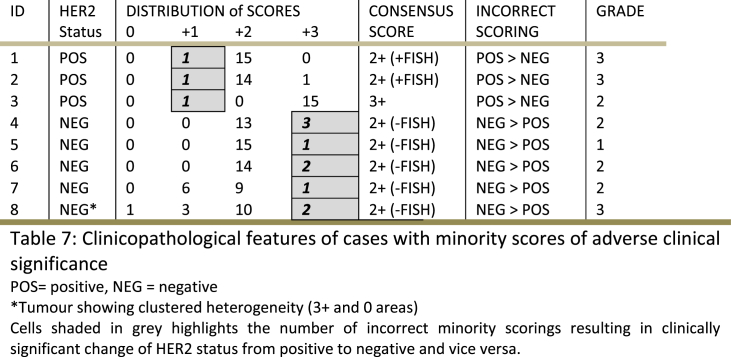
Clinicopathological features of cases with minority scores of adverse clinical significance POS = positive, NEG = negative *Tumour showing clustered heterogeneity (3+ and 0 areas) Cells shaded in grey highlights the number of incorrect minority scorings resulting in clinically significant change of HER2 status from positive to negative and vice versa.

**Fig. 2c:**
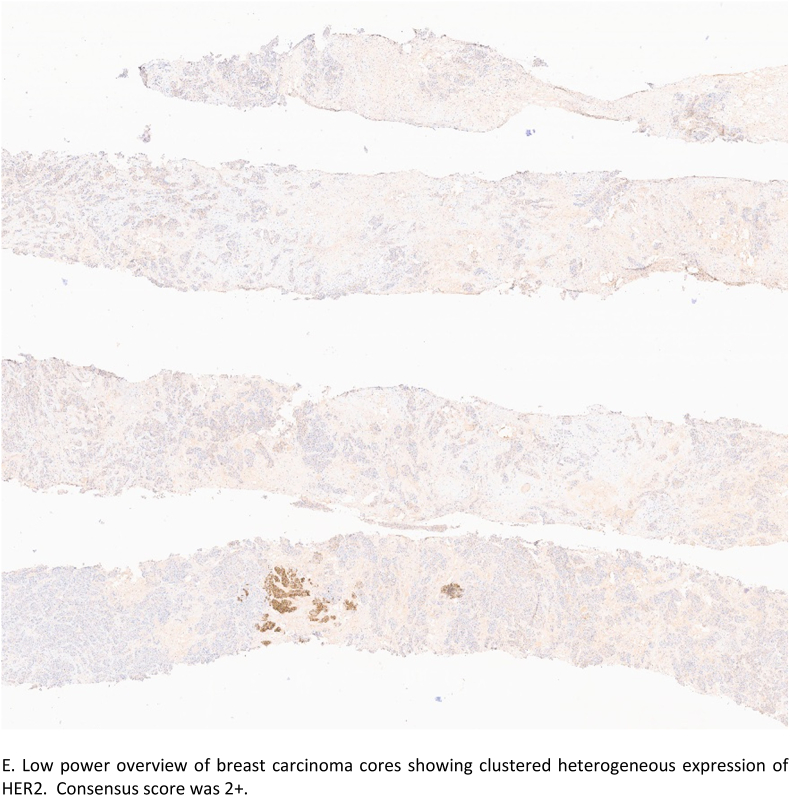
E. Low power overview of breast carcinoma cores showing clustered heterogeneous expression of HER2. Consensus score was 2+.

**Fig. 2d:**
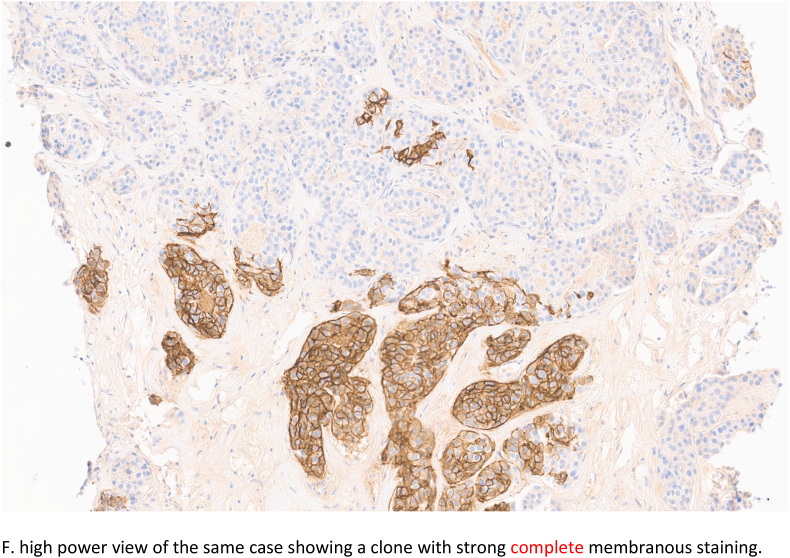
F. high power view of the same case showing a clone with strong complete membranous staining.

Fleiss Multi-rater kappa of overall agreement (OA) and among scoring categories was calculated, Table 8. Improvement in kappa of OA agreement and individual agreement was obtained with clustering scores 1+ and 2+ categories (1/2), Table 8.

**Table 8:**
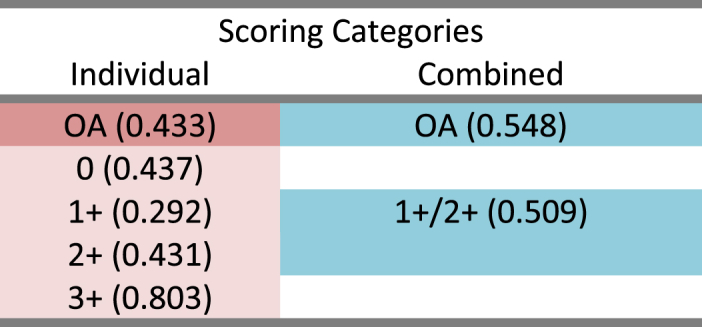
Fleiss multi-rater kappa of agreement, among individual and combined categories. OA: overall agreement.

### 5.2 Inter-observer and consensus observers’ agreement

Cohen's Kappa was calculated to assess inter-observer agreement between each two of the scoring pathologists as well as between each pathologist and the consensus score. This has been investigated in the whole cohort and in the HER2-low group (Table 9). In the whole cohort, inter-observer agreement was predominantly moderate (46%) to substantial (41%), while it was fair (40.7%) to moderate (30%) in the HER2-low group. Similarly, consensus-observer agreement was substantial (50%) to almost perfect (19%) in the whole cohort, while it was moderate (43.75%) to substantial (25%) in the HER2-low group (Table 10).

**Table 9:**
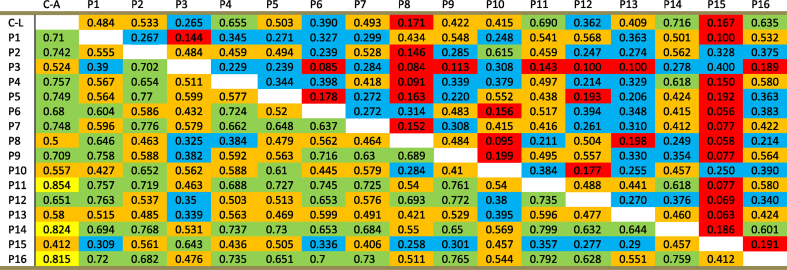
Cohen's weighted kappa for pairwise agreement between each rater's score and the consensus score, in all HER2 IHC categories and in the HER2-low group. The bottom left triangular half below the equatorial blank cells represents values of the agreement among the whole cohort of cases (n = 50), while the opposite top right triangle represents the HER2-low cases (n = 36). (*C*-A): Consensus score of the whole cohort; (*C*-L): Consensus score of the HER2-low cohort. Cell shading colours reflect kappa levels of agreement as follows; yellow for almost perfect agreement (0.81–1), green for substantial agreement (0.61–0.8), orange for moderate agreement (0.41–0.6), blue for fair agreement (0.21–0.4), and red for poor agreement (0–0.2).

**Table 10:**
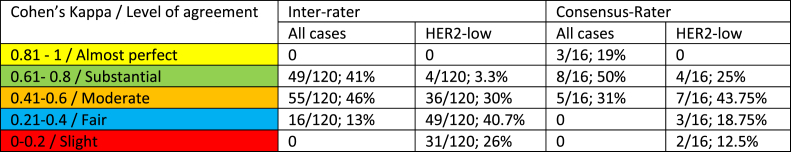
Cohen's weighted kappa for pairwise agreement between.

## 6 Discussion

This is the first set of data from the UK and Ireland on the consistency of pathologists reporting of the HER2-low and ultralow categories of invasive breast carcinomas. In addition to the categorisation of the HER2 IHC into the standard scores of 0, 1+, 2+ and 3+, pathologists detailed the parameters they based their scores on, including whether the membranous staining was circumferential or not, intensity of staining and the percentage of membranous staining expressed as < or ≥10%. These parameters follow the recently updated UK HER2 reporting guidelines that also recognize HER2-low tumours as a new entity [7]. The majority of tumours (90%) were accurately classified. However, a small proportion (10%) remained challenging. Reasons for discordance were predominantly due to difficulties in estimating percentage expression around the 10% cut off value. HER2 cytoplasmic staining rendered the assessment of faint membrane positivity rather tricky and heterogeneity of HER2 expression resulted in difficulties in estimating the percentage of expression. Cohen Kappa statistics showed lower inter-observer and observer-consensus agreement in the HER2-low group compared with the whole four standard HER2 IHC scoring categories. This lower consistency for diagnosing the HER2-low group requires further work to refine the categorisation and improve concordance.

Tumour heterogeneity has been highlighted as an important issue causing difficulties in HER2 IHC and FISH interpretation and leading to uncertainty on treatment decisions. Three patterns of heterogeneity have been described including clustered (two distinct patterns of positive and negative expression), mosaic, and scattered heterogeneity [12]. One of the tumours with discordant scoring among the non-majority diagnoses showed a clustered pattern of expression (small 3+ focus within a majority negative score 0 tumour), Fig. 2c, Fig. 2dE and F. It is of note that further sampling from a metastatic axillary lymph node showed only a HER2 0 profile. Following multidisciplinary discussion, the tumour was regarded and managed as HER2 negative (TNBC) in view of the very focal expression and the patient received neoadjuvant chemotherapy (NACT). Testing of the residual carcinoma post NACT revealed TNBC with a HER2 IHC score of 0. HER2 heterogeneity is known to be more prevalent in the borderline and negative groups and hence will be represented at a higher frequency in the HER2-low tumours. This adds to the complexity of HER2-low scoring and may influence the correct categorisation of such tumours.

The type of specimen, i.e. biopsy versus excision, has recently been shown to be relevant in the assessment of HER2 low cancer. In a Chinese series of 5610 paired specimens, a discordance rate of 17.63% was found between core and excision HER2 low assessment. This was largely presented by cases diagnosed as HER2-low on surgical excision following a core biopsy assessment as negative, score 0 (n = 530, 9.4%). The reverse also occurred with 387 cases (6.9%) switching from a HER2 0 to HER2-low status [13]. In routine practice, HER2 is tested primarily on diagnostic core biopsies and hence the assessment of breast cores in the present study.

The HER2-low group represents a considerable proportion (40–50%) of all breast cancers and includes both hormone receptor positive and negative carcinomas with more representation of the former [[14], [15], [16]]. A real world UK and Ireland web based biomarker audit showed that HER2 1+ tumours accounted for 32.2% of all breast cancers, and the HER2 equivocal (2+) category for 12.9%, with 72.1% of the latter FISH non-amplified. This corresponds to a rate of 41.5% HER2-low breast cancers [17]. In a study of 281 consecutive breast cancers, the HER2-low group represented 31% of tumours and was more common in the ER positive group compared with ER negative tumours (33.6% vs 15%, *p* = 0.017). Those tumours were predominantly of ‘ductal’ type [18]. Following on from the National Comprehensive Cancer Network (NCCN) guidelines version 3.2023 [19], other national and international guidelines are likely to include the HER2-low category in management pathways and therefore standardised diagnostic criteria should be available for pathologists to ensure consistency of diagnosis.

Concordance studies of HER2-low scoring among pathologists were recently identified as an unmet research need by Baez-Navarro and colleagues [20]. Awareness of the recent advances in therapeutic options and the requirement to correctly identify the HER2-low group of breast tumours is particularly relevant as the boundaries between 0 and 1+ scores, as highlighted in the current study, are less clear. In the current state of knowledge, it is not known whether pathologists will also be required to identify tumours with HER2 ultralow expression. Initial data from the DAISY trial (Study of DS-8201a, an Antibody Drug Conjugate for Advanced Breast Cancer Patients, With Biomarkers Analysis (DAISY) (ClinicalTrials.gov Identifier: NCT04132960), suggest that patients with tumours that display very low levels of HER2 expression (currently regarded as score 0) may still benefit from T-DXd therapy. If this is proven, reversion to the ASCO/CAP 2007 criteria of IHC score 0 confined to tumours with complete absence of HER2 expression (rather than the current 10% cut off) would be appropriate [20]. We have shown that combining scoring categories resulted in a higher concordance among pathologists and will meet the clinical need. This will undoubtedly be guided by the clinical requirements for determining patient eligibility for treatment with anti-HER2 ADCs and awaits evidence from current ongoing trials.

Recently, Baez -Navarro et al. assessed the concordance of HER2 scoring among 16 pathologists in two rounds. In round one, absolute agreement on all categories was reached in a minority of cases (4.7%). This compares with 6% in the current study. Best absolute agreement (74.3%) was reached when the HER2 0 category (using the 2007 ASCO/CAP guidelines) was compared to a combined group of HER2 1+/2+ (i.e., no staining vs any staining that is not 3+) [9]. Our study confirmed the above data where clustering of score 0 against the rest of the IHC scores combined resulted in increased both absolute and high agreement to 86% (compared to 52%). Combining scores 1+ and 2+ also resulted in improved concordance with higher percentages of absolute and high agreement to 84%. While the use of the term HER2-low by pathologists may not be currently required as per the recent ESMO consensus [21], it is essential that pathologists distinguish between HER2 scores 0 and 1+ in their report. An unqualified HER2 negative statement, that was previously acceptable for primarily identifying the HER2 positive cancers, is no longer justified. Data from the current study also supports that the use of the 2007 guidelines for the definition of HER2 0 category as complete absence of membranous staining would improve the consistency of pathologists scoring. This group lacking any evidence of HER2 expression has been recognized and referred to as “HER2-null”. The decision as to whether this latter term should be used in conjunction with the HER2-ultralow category awaits the results of the DB-06 trial [21].

The current study cohort of breast tumours was stained using the 4B5 Ventana IHC assay. Differences among HER2 staining platforms exist and the proportion of tumours regarded as HER2-low may vary according to the assay used. A recent comparison of 119 breast cancers stained by the Dako polyclonal HercepTest and the Ventana monoclonal 4B5 assay revealed that the former classified more cases as HER2 2+ [22], including tumours with HER2 amplification and tumours that were non-amplified (HER2-low). However, an earlier study of 500 primary and metastatic breast cancer tested the concordance of the two tests and showed the proportion of HER2 1+/2+ scores to be 28% and 11.6% by the 4B5 and HercepTest respectively, with several tumours designated as HER2 0 by the HercepTest, classified as 1+/2+ (i.e., HER2-low) by the 4B5 assay. All tumours that scored 0 by the HercepTest were also scored 0 by the 4B5 assay [10]. In a series of 205, mostly HER2 score 2+ tumours, stained by the HercepTest at 14 Spanish centres and tested centrally by FISH, agreement was only low to moderate [23]. The number of tumours that were FISH negative (i.e., HER2-low) increased with a decrease in the number of coincident observers but the opposite was found for the FISH positive cases. Possible reasons for disagreement, proposed by the authors, included subjective assessment, pre-analytical conditions of samples, criteria used and observer experience [23].

Data on the prognostic significance of HER2-low tumours are conflicting. Xu et al. analysed 777 non-HER2 positive breast cancers and found no prognostic differences between the HER2-2 low and the HER-2 negative group. The hormone receptor HER2-low group had a better prognosis [24]. PAM50 date from 3689 HER2-low and HER2 score 0 breast cancers revealed that the HER2-low tumours were predominantly of the luminal type compared with the TNBC (65.4% VS 36.6% respectively) [25].

Similarly, a large retrospective study of breast tumours from 5235 patients, that included 2917 HER2-low tumours, did not identify any specific histopathological features associated with this HER2 profile^17^. The expression of hormone receptors was significantly higher in HER2-low compared with the HER2 0 tumours (p < 0.001) [26] but there was no significant difference in disease-free or overall survival between hormone receptor positive and hormone receptor negative HER-2 low tumours. These findings imply that HER2-low breast cancer is not a distinct biological entity. Others, however, have proposed that, particularly in the neoadjuvant chemotherapy setting, that HER2-low tumours can be considered a “new subgroup of breast cancer by standardised IHC, distinct from HER2-zero tumours” highlighting a lower pathological response rate in hormone receptor positive lesions compared to HER2 negative lesions and differences in prognosis [27]. Clearly this potential group of invasive breast cancers requires further research. A recent study of 351 patients including 189 with HER2 low breast cancers, reported that the latter had better survival compared with the HER2 0 group [28].

Strengths of the current study include that participants, Professional Clinical Advisors for the NHS Breast Screening Programme, are experts in breast pathology morphological diagnosis and HER2 immunohistochemistry assessment. Their roles include supporting delivery of high quality of breast cancer service and providing guidance to pathologists in various regions of the UK and Ireland. These data will inform education and training of breast and molecular pathologists in both countries. Another strength is the robust IHC staining and FISH protocols performed at a large UK molecular pathology service which is also a regional referral centre for HER2 immunohistochemistry and FISH testing. In addition, all tumours were stained using the Ventana PATHWAY anti-HER2/neu (4B5) Rabbit Monoclonal Primary Antibody test. It is the most commonly used HER2 IHC assay in the UK, the test used for the DESTINY-Breast 04 trial [4] and an approved companion diagnostic for HER2-low assessment by the U.S. Food and Drug Administration (FDA). The use of digital pathology is a strength that ensured that all scorers assessed the identical slides but can also be a limitation for pathologists who are less familiar with using digital platforms for scoring. A limitation of this study is the relatively small number of tumours (50) included. Whilst all these cases were core biopsy specimens, this is the specimen that is assessed for HER2 testing in the UK. This may not reflect the examination of HER2 status on breast cancer excisions. Of particular note, the cases in this study were enriched for HER2-low expressors and selected to predominantly assess the concordance at the lower end of the HER2 staining spectrum; in other words, it must be noted that the concordance presented here does not necessarily represent the agreement for overall HER2 testing for all cases.

This current study highlights that a proportion breast cancers can be challenging to designate as immunohistochemistry score 1+ versus 0 even for expert breast pathologists. We recommend double soring of those cases at the border between the two categories and/or discussion with colleagues to reach a consensus. Pre-analytical factors such as appropriate fixation, careful attention to the quality and sensitivity of HER2 immunohistochemically staining and use of appropriate controls are also essential. This will be facilitated by the enrolment in relevant laboratory quality assurance programmes. Recently, the United Kingdom National External Quality Assessment Service (UK NEQAS) has launched an educational HER2-low technical and interpretive pilot scheme and presented findings of the first round. Results have shown poor concordance (<50%) in HER2-low laboratory staining and scores when compared with expert pathologists using a well validated assay [29]. Continuous education and raising awareness among pathologists will improve concordance and reporting accuracy.

Digital pathology and artificial intelligence (AI) algorithms have been suggested as promising applications for HER2 IHC and FISH assessment [30]. AI algorithms for HER2 scoring are promising but can only be implemented if the gold standard pathologist reporting can be standardised for an AI algorithm calibration. Furthermore, other techniques such as quantitative immunofluorescence [31] and *in vitro* diagnostic RNA based tests for the assessment of HER2 status, particularly at the low expression level, have been developed [32,33]. These may provide more accurate and reproducible categorisation of the HER2-low tumours compared with IHC or used as confirmatory tests in challenging cases. Further studies comparing various methods of HER2 assessment against tumour response are required to inform decision-making and therapeutic strategies.

## Declaration of competing interest

EP has received honoraria from Roche, Novartis and AstraZeneca for lectures and participating in advisory committees. SP has received honoraria as speaker and advisory board member for Exact Sciences. AMS has received honoraria as speaker and advisory board member for Exact Sciences, Veracyte, Roche, Hologic, Diaceutics and AstraZeneca.
